# A Comparative Study of the Dynamics and Diversity of *Bdellovibrio* and Like Organisms in Lakes Annecy and Geneva

**DOI:** 10.3390/microorganisms10101960

**Published:** 2022-09-30

**Authors:** Jade A. Ezzedine, Mathilde Scheifler, Yves Desdevises, Stéphan Jacquet

**Affiliations:** 1 Université Savoie Mont-Blanc, INRAE, CARRTEL, 74200 Thonon les Bains, France; 2 Laboratoire de Physiologie Cellulaire et Végétale, CNRS, CEA, INRAE, IRIG, Université Grenoble Alpes, 38058 Grenoble, France; 3 Sorbonne Université, CNRS, Biologie Intégrative des Organismes Marins, BIOM, Observatoire Océanologique, F-66650 Banyuls-sur-Mer, France

**Keywords:** lake, bacteria, *Bdellovibrio* and like organisms, diversity, dynamics

## Abstract

*Bdellovibrio* and like organisms (BALOs) are obligate bacterial predators of other Gram-negative bacteria. Here, we used quantitative PCR (qPCR) and recently developed specific primers which target the 16S rRNA gene to explore the abundance and distribution of three families of BALO belonging to the Oligoflexia class (i.e., *Bdellovibrionaceae*, *Peredibacteraceae* and *Bacteriovoracaceae)* over one year in the epilimnion and hypolimnion of Lakes Annecy and Geneva. *Peredibacteraceae* was the dominant group at all sampling points except at the bottom of Lake Geneva, where *Bdellovibrionaceae* was found in higher number. In addition, the abundance of BALOs increased significantly during the warmer months. Using high-throughput sequencing (Illumina Miseq), hundreds of OTUs were identified for *Bdellovibrionaceae* and *Peredibacteraceae*. Phylogenetic analysis suggests that *Bdellovibrionaceae* are more diverse than *Peredibacteraceae* and that some OTUs belong to new species of *Bdellovibrionaceae*. We also found that dominant OTUs were present simultaneously in the two lakes, while some others were specific to each lake, suggesting an adaptive pattern. Finally, both abundance and diversity of BALOs were poorly associated with abiotic factors except temperature, suggesting the importance of studying biotic relationships, assumed to play a greater role than physico-chemical variables in BALOs’ dynamics and distribution.

## 1. Introduction

Among microorganisms inhabiting aquatic systems, a large variety of micropredators remains poorly known. These micropredators belong to various groups (i.e., metazooplankton, ciliates, flagellates, fungi, bacteria) and are facultative and/or obligatory hunters of bacteria [1]. Amongst predatory heterotrophic bacteria, Myxobacteria are facultative hunters but predate on other bacteria when food resources become scarce [2]. By contrast, there is one group that consists of obligate predators of Gram-negative bacteria referred to as “*Bdellovibrio* and Like Organisms” (named BALOs thereafter) which belong to the Oligoflexia and Alpha-proteobacteria.

BALOs are reported to be widely distributed in natural and man-made habitats [3]. Since these small bacterial hunters can be abundant in favorable situations (typically where prey are abundant), they have been proposed as “population balancer” [4,5] and a “driver of bacterial alpha diversity” [6]. Indeed, BALOs could limit competition and favor rare taxa by curbing dominant species. Additionally, this functional group of bacteria has been reported to exert strong effects on bacterial pathogens [7]. Thus, numerous applications using BALOs are in development [8], in particular in medicine [4,9], aquaculture [10,11], agriculture [12,13] and in the food industry [14,15] with the goal to reduce or eliminate pathogens. However, these bacteria remain largely underexplored and their ecology (particularly in natural aquatic ecosystems) is still poorly documented compared to other bacterial groups.

The goal of the present study is to shed light on the dynamics of BALOs in natural freshwater environments. However, unlike our previous studies that involved short term dynamics, this one is based on a full-year sampling of two different water column layers (e.g., the epi- and hypolimnion) of Lakes Annecy (France) and Geneva (Switzerland/France). These two lakes have been the object of many studies in recent decades, and they belong to the OLA lake (monitoring) observatory (https://www6.inrae.fr/soere-ola (accessed on 23 march 2020)). It is noteworthy that Lake Geneva is the largest natural and deep lake in Western Europe, while Lake Annecy is the second largest in France. In both lakes, BALOs were detected in a previous study and likely to be a functional bacterial group of interest (24). Using the recent BALOs classification [16,17] and recently designed pairs of primers by ourselves [18], we focused on three families of BALOs from the Oligoflexia group: *Bdellovibrionaceae*, *Peredibacteraceae* and *Bacteriovoracaceae*. We explored the *Bdellovibrionaceae* and *Peredibacteraceae* diversity using high-throughput sequencing. The abundance and distribution of *Bdellovibrionaceae*, *Peredibacteraceae* and *Bacteriovoracaceae* were assessed using qPCR. We investigated the following questions: Are these BALOs abundant in the studied ecosystems and at which depth? Which BALOs dominate these ecosystems? What variables may explain the distribution and structure of these bacterial predators?

## 2. Materials and Methods

### 2.1. Study Sites, Sampling and Environmental Descriptors

An annual water sampling, once in a month, was carried out in two freshwater ecosystems for which long-term ecological surveys exist. The locations investigated were Lakes Geneva (46°27′9.72 N–6°35′19.4 E) and Annecy (45°52′23.42 N–6°7′45.78 E). In Lake Geneva, the SHL2 point corresponds to the CIPEL monitoring site (established in 1962) [19], and in Lake Annecy, the GL site has been monitored by the SILA since 1966 [20]. The CIPEL and SILA mandate the OLA observatory for data collection and analysis [21]. Sampling of Lakes Geneva and Annecy took place from February 2018 to January 2019. BALOs were mainly investigated in the epilimnion and hypolimnion. The epilimnion layer is known to be relatively homogeneous, warm, well lighted and rich with autotrophs, where the hypolimnion is almost deprived of light and rich with heterotrophic bacteria. To coincide with these layers, the sampled depths were 2.5 and 50 m for Lake Geneva, and 3 and 45 m for Lake Annecy. However, we are also reporting the results obtained at the 200 m depth for Lake Geneva, where a high diversity of prokaryotic OTUs were found [22]. Note that the comparison with Lake Annecy is not possible since its maximum depth is 82 m.

Two replicates of a 2 L volume of water from each sampled depth were serially filtered over 5 and 2 µm polycarbonate (PC) filters to remove impurity and bigger microorganisms such as eukaryotic grazers. Then, one liter of the <2 µm filtered water was filtered on a 0.2 µm PC filter. Then, 0.2 µm filters were kept and stored at −80 °C until DNA extraction. In parallel, multiple physico-chemical descriptors such as calcium, magnesium, sodium, potassium, phosphorus, carbon, nitrogen, oxygen, pH, temperature, chlorophyll, conductivity, etc., were measured at each site by OLA’s observatory, according to standardized protocols [21] (https://si-ola.inra.fr/si_lacs/ (accessed on 23 march 2020)). Only significant variables were retained for analysis, as explained below.

### 2.2. DNA Extraction

The two replicates of the 0.2 µm filters were subjected to DNA extraction using a homemade protocol that have been shown to be efficient in previous studies [18,23,24,25]. First, all samples were centrifuged for 3 min at 6000× *g* and 4 °C. The supernatant was discarded. Second, 300 µL of TE buffer (TRIS: 1 M—pH 8, EDTA: 0.5 M—pH 8) was added to the pellet. Next, a lysis step was performed by adding 200 µL of lysis solution (TRIS: 1 M—pH 8, EDTA: 0.5 M—pH 8 and sucrose: 0.7 M). After that, a thermic shock was performed at −80 °C for 15 min, and samples were immediately thawed into a block heater at 55 °C for 2 min. Then, 50 µL of 10% sodium dodecyl sulfate (SDS) as well as 10 µL of proteinase K (20 mg/mL) were added. Samples were then incubated at 37 °C for 1 h with gentle stirring, and placed in a heating block at 55 °C for 20 min. After a quick centrifugation step (13,000 rpm at 4 °C for 3 min), the supernatant was collected. Then, 50 µL of sodium acetate (3 M—pH 5.2) and 1.5 µL of GenEluteTM-LPA (Sigma-Aldrich, Saint Louis, MO, USA, 25 µg/µL) were added. Next, one volume of isopropanol was added. An overnight precipitation was performed, then the tubes were centrifuged for 10 min at 12,000× *g* and 4 °C. Following this step, two rounds of ethanol (80%) washing were carried out to clean the DNA pellet. The remaining ethanol was evaporated using a SpeedVac for 20 min. Finally, 30 µL of TE was added, and samples were incubated, at 37 °C, for 1 h to let the pellet gently dissolve into the TE buffer. DNA concentration was measured using a NanoDrop 1000 spectrophotometer. For DNA concentration superior to 25 ng/µL, a dilution was performed. All DNA preparations were stored at −20 °C until analysis.

### 2.3. qPCR Standard Curves

One replicate of the 0.2 µm PC filters was used to measure the abundances of *Bdellovibrionaceae, Peredibacteraceae*, *Bacteriovoracaceae* in Lakes Geneva and Annecy, at all sampled depths. Quantitative PCR (qPCR) was conducted using a Rotor-Gene Q machine (Qiagen). The set of primers used to measure BALOs abundance were Bd 347F–549R [26] (*Bdellovibrionaceae*), Per 627F–696R [18] (*Peredibacteraceae*) and Bx 421F–482R [16] (*Bacteriovoracaceae*). All primers target the 16S rRNA gene. Standards were prepared using identified clones as stated in our previous study [18]. In brief, plasmids were extracted and purified using NucleoSpin Plasmid kit (Macherey-Nagel, Allentown, PA, USA) according to the manufacturer’s instructions. Plasmids were then digested with BamH I restriction enzyme following manufacturer’s instructions. The digested plasmid concentrations were measured using Quant-iT PicoGreen ds DNA Reagent kit (Invitrogen, Waltham, MA, USA) and fluorescence was read using a plate reader Fluoroskan Ascent FL. The number of copies for each BALOs clone was calculated using the following formula [27]:

$$Number\;of\;copies=\frac{DNA\;concentration}{\left(Insert\;size+Plasmid\;size\right)\times 660}\times 6.02\times 10^{23}$$

Then, serial dilution was conducted from 10^9^ to 10^0^ copies. Diluted DNA from 10^7^ to 10^0^ were used in duplicate and amplified by BALOs qPCR set of primers to constitute the standard curve. Two controls were added each time. Lakes samples were integrated in the same run. The qPCR mixture volume was 25 µL and consisted of (final concentration): 1 X Master Mix (QuantiTect SYBR Green PCR kit, Qiagen, Hilden, Germany), 0.3 mg mL^−1^ of BSA, 0.2 µM of forward and reverse primers and 1 µL of template DNA (25 ng µL^−1^). The program used was as follows: 95 °C—15 min, 40 × (95 °C—45 s, 60 °C—45 s, 72 °C—45 s), + 1 °C every 5 s from 60 to 95 °C. The standard curve parameters for *Bdellovibrionaceae* were R^2^ = 0.99916 and efficiency = 0.95. The threshold was set to 0.02. In the same logic, *Peredibacteraceae* standard curve parameters were R^2^ = 0.999762, efficiency = 0.91 and threshold set to 0.015. For *Bacteriovoracaceae* R^2^ = 0.99784, efficiency = 0.93, and threshold set to 0.02. For all environmental samples amplified with BALOs primers, those that failed to amplify or were outside (lower) the standard curves were not considered in the analysis. Here, we worked with copy per milliliter instead of cell number per milliliter since the copy number of 16S rRNA gene is only reported for very few species of BALOs. In the NCBI genome section, *Peredibacter* sp. and *Bdellovibrio bacteriovorus* ASM69160V1 are considered to have one copy of 16S rRNA while *Bacteriovorax stolpii* ASM687460V1 have 2 copies of 16S rRNA.

BALOs abundances were obtained in copy per reaction and were transformed to copy per milliliter using the following formula:

$$Copy\;per\;milliliter=\frac{\frac{Copy\;per\;reaction}{Dilution\;factor\;for\;25{\;\mathrm{ng}\;µL}^{-1}}\times DNA\;elution\;volume}{Filtered\;volume\;on\;0.2\;µm}$$

### 2.4. PCR and Next-Generation Sequencing

One replicate of the 0.2-µm PC filters was used to extract DNA that was amplified and sequenced using BALOs specific primers that targeted the 16S rDNA gene. The run consisted of 96 samples from Lakes Geneva and Annecy from two depths, i.e., surface vs. bottom (2.5 and 200 m for Lake Geneva and 3 and 45 m for Lake Annecy). The specific primers used for *Bdellovibrionaceae* were Bd-F186 and Bd-R481 and for *Peredibacteraceae,* Per-F1024 and Per-R1349 [18]. Prior to sequencing, samples were amplified by polymerase chain reaction (PCR) using a combination of tags of 8 bases long [22,25] attached to the specific set of primers. Hence, each sample could be discriminated by the tagging of forward and reverse primers. Total PCR mixture volume was 50 µL and consisted of (final concentration): 1 U buffer, 0.4 mM dNTP, 2 mM MgCl_2_, 0.4 mg mL^−1^ bovine serum albumin (BSA) and 1 U Biotaq DNA polymerase (Bioline, Cincinnati, OH, USA). A unique combination of tagged primers (0.2 µM) was added in a second step to each sample. In addition, 1 µL of template DNA (25 ng µL^−1^) was added. Negative controls were included and the PCR program was as follows: 95 °C—2 min, 30 × (94 °C—30 s, 58 °C—30 s, 72 °C—30 s), with a final extension step at 72 °C for 5 min. Agarose gel (1.5%) analysis was performed for verification of the PCR products. Primer dimers were present so we did not measure the quantity of amplified DNA in each sample. The amount of DNA per sample is therefore not equivalent and is subject to how the PCR reaction has amplified each sample. Then, four pools (no equimolar concentration) were constituted: amplified sequences with *Bdellovibrionaceae* primer pair from Lake Geneva (Bd- Geneva), the same for Lake Annecy (Bd- Annecy), amplified sequences with *Peredibacteraceae* set of primers for Lake Geneva (Per- Geneva), and the same for Lake Annecy (Per- Annecy). These four pools were checked again on agarose gel and under UV light, expected band was cut using a sterile scalpel. The excised band was purified using the Illustra GFX Gel Band Purification Kit following the manufacturer’s instructions. Then, each pool was measured using the Quant-iT PicoGreen dsDNA Reagent kit (Invitrogen) and fluorescence was read using a plate reader Fluoroskan Ascent FL to make a single equimolar pool containing 1000 ng DNA. The final pool was sent to the GATC-Eurofins platform for DNA sequencing using Illumina Miseq 250 bp paired-end technology (6M reads package).

### 2.5. Bioinformatic Pipeline

R1 and R2 fastq files were processed using Frederic Mahé’s pipeline found at https://github.com/frederic-mahe/swarm/wiki/Fred’s-metabarcoding-pipeline (accessed on 2 January 2020). Briefly, the pipeline uses several programs such as Vsearch [28], Cutadapt [29], Swarm and Stampa [30]. All default parameters of the pipeline were left unchanged unless stated otherwise. OTUs were created using Swarm with “d = 1”. All OTUs were taxonomically assigned with the arb-SILVA database release number 138 [31]. OTU tables were filtered by removing chimera sequences, singletons, sequences with less than 90% identity to arb-SILVA database and sequences with a quality score inferior to 0.0002. After the application of the aforementioned filters, the number of sequences for *Bdellovibrionaceae* dropped to 6830 in Lake Geneva (14% loss) and 1054 in Lake Annecy (26% loss). As for *Peredibacteraceae* sequences, they reached 41,174 in Lake Geneva (6% loss) and 55808 in Lake Annecy (4% loss). The overall number of sequences went from 111,089 to 104,866 (6% loss). The rarefaction curve (Figure S3) suggests that the community of BALOs were very different from one sample to another and that a plateau was not reached for all samples. This may be attributed to low sampling depth or the use of specific primers for a few species. Rarefaction was not applied to the samples in order to avoid losing rare BALOs taxa. Therefore, the data was normalized by transforming it into relative abundance.

### 2.6. Statistics

For the abundance data (copy mL^−1^), the values were transformed using log(x). All graphs were built via ggplot2 [32] using the transformed values. The “vegan” package [33] was used to analyze the link between BALOs and environmental variables. First, a DCA (detrended correspondence analysis) was performed on Lakes Geneva and Annecy data (DCA axis lengths < 3). The test determined that RDA (redundancy analysis) was the adequate choice for the analysis [34]. First, “corrplot” package [35] was used to visually select relevant environmental variables, then variance inflation factors (VIF) were calculated and only variables with VIF < 10 were retained. The RDA significance was checked using a 999 permutations ANOVA test.

For OTUs, non-metric multidimensional scaling (NMDS), permutational multivariate analysis of variance using distance matrices (Adonis), canonical correspondence analysis (CCA) and rarefaction curves were performed using the R vegan package [33]. Here, CCA were performed since the DCA was >3. In addition, the significance of the CCA was tested using ANOVA, and variance inflation factors (VIF) were calculated for environmental variables and only those with VIF < 10 were retained. Venn diagram were drawn using the online tool found at http://bioinformatics.psb.ugent.be/webtools/Venn/ (accessed on 22 February 2020).

### 2.7. Flow Cytometry Analysis

We used a FACSCalibur flow cytometer (BD BioSciences, Franklin Lakes, NJ, USA) to determine the total prokaryote abundance. Each water sample was thawed at ambient temperature, then 2.5 µL was added to 245 µL filtered (<0.02 µm) TE buffer and 2.5 µL of SYBR Green I (diluted 10,000 times). The sample was then heated for 10 min at 75 °C before the FCM analysis [36]. “List-mode” files were exported and analyzed using CYTOWIN [37]. The analysis provided information on prokaryote-like particles (PLPs).

## 3. Results

### 3.1. BALOs Abundance, Distribution and Dynamics

**Figure 1:**
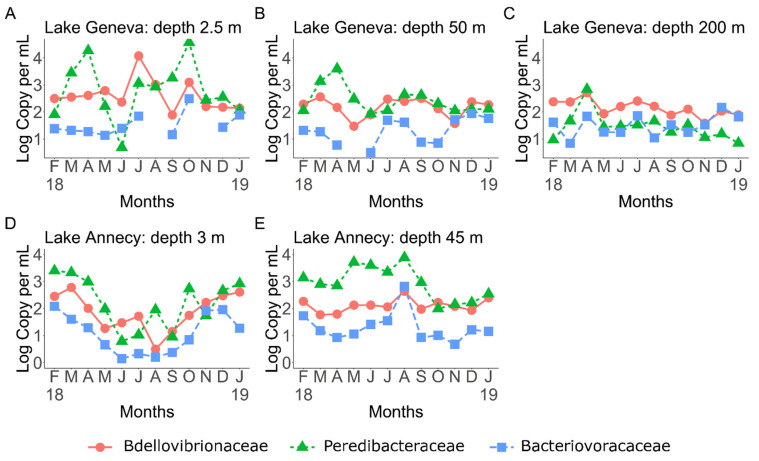
Abundance (copy per mL) distribution and dynamics of the three BALOs families, *Bdellovibrionaceae, Peredibacteraceae* and *Bacteriovoracaceae*, over one year (February 2018 to January 2019) in Lake Geneva (at 2.5 m Figure S1, 50 m Figure S2 and 200 m Figure S3) and Lake Annecy (at 3 m Figure S3 and 45 m Figure S5). The x-axes indicates the months, each abbreviated by the first letter (F: February; M: March; A: April; M: May; J: June; J: July; A: August; S: September; O: October; N: November; D: December; J: January).

Both abundances and distribution of BALOs varied across the examined lakes (Figure 1). Because some BALOs were not detected or well amplified, we focused on samples from which enough material could be obtained. The abundance of BALOs fluctuated monthly and with depth. We found that *Bdellovibrionaceae* and *Peredibacteraceae* were more abundant than *Bacteriovoracaceae*. *Peredibacteraceae* seemed to be favored in the epilimnion of Lake Geneva (Figure 1A), while in Lake Annecy, they were more abundant in the hypolimnion (Figure 1E). *Bdellovibrionaceae,* less dominant than *Peredibacteraceae,* were more present in the hypolimnion of Lake Geneva (Figure 1C). Finally, *Bacteriovoracaceae* were less detected in both lakes.

Overall, *Peredibacteraceae* dominated in terms of abundance (with concentrations up to 71,700 and 31,137 copy mL^−1^ for Lakes Geneva and Annecy, respectively), followed by the *Bdellovibrionaceae* (reaching up to 20,944 and 3856 copy mL^−1^ for Lakes Geneva and Annecy, respectively) (Figure S1). By contrast, *Bacteriovoracaceae* were in general in low abundance or not detected. BALOs were more abundant in surface waters (reaching up to 80,550 copy mL^−1^) for Lake Geneva. However, it was the opposite in Lake Annecy, in which the abundance at 45 m could reach 26,153 copy mL^−1^ vs. 10,089 copy mL^−1^ at the surface. BALOs’ abundances in the two studied ecosystems at all depths and throughout the year can be found in Table S1.

The abundance of BALOs varied over the year and across lakes, showing various dynamics (Figure S2). In Lake Geneva, the seasonal patterns observed for *Bdellovibrionaceae* were rather similar at 2.5, 50 and 200 m, except in July and October where higher values were reached at 2.5 m (Figure S2A). The same type of pattern was observed for the *Peredibacteraceae* but for different months, i.e., April and October (Figure S2B), with an important decrease observed in June. The *Bacteriovoracaceae* dynamics were more difficult to interpret, with relatively constant abundances along the year (Figure S2C). For Lake Annecy, BALOs abundance declined significantly in surface during winter and spring and increased again in August, especially for the *Peredibacteraceae*. The opposite trend was observed at 45 m.

**Figure 2:**
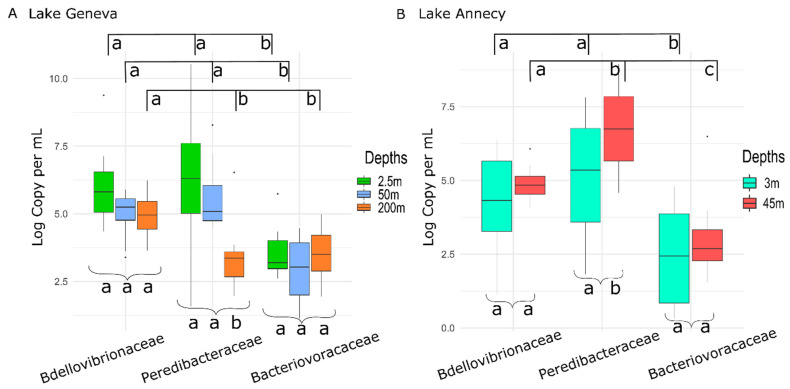
Mean values of the three BALOs at the studied depths of Lakes Geneva and Annecy (*n* = 12 for each boxplot). Brackets are used to illustrate the comparison for a family of predators between the different depths studied. Square brackets display the comparison between the predators for a similar depth. Letters represent the statistical significance (*p* < 0.05) of differences between the studied groups.

When looking at the mean abundance of BALOs at each depth during the 12 months of sampling (Figure 2), both *Bdellovibrionaceae* and *Bacteriovoracaceae* were not significantly different between depths in Lake Geneva (*p*-value = 0.06 and 0.95) (Figure 2A). An opposite trend was observed for the *Peredibacteraceae* (*p*-value = 0.0004) with a significant difference between surface or intermediate waters and 200 m depth (2.5–200 m *p*-value = 0.0003; 50–200 m *p*-value = 0.003) and significantly higher abundances in the upper layer. When comparing *Bdellovibrionaceae, Peredibacteraceae* and *Bacteriovoracaceae,* the mean abundance at 2.5 m of the latter was significantly different (i.e., less abundant) from the mean abundance of the two others (*p*-value = 0.0017 and 0.0008). By contrast, there was no difference between the mean abundances of *Bdellovibrionaceae* and *Peredibacteraceae* at 2.5 m (*p*-value = 1). At 50 m, similar results as above were observed (*p*-value = 0.001 between *Bacteriovoracaceae* and *Bdellovibrionaceae*; *p*-value = 0.0001 between *Bacteriovoracaceae* and *Peredibacteraceae*). At 200 m the mean abundances between *Bdellovibrionaceae* and the two other families were also significantly different (i.e., more abundant, *p*-value = 0.0004 between *Bdellovibrionaceae* and *Peredibacteraceae; p*-value = 0.014 between *Bdellovibrionaceae* and *Bacteriovoracaceae*). However, the mean abundance was not different between *Peredibacteraceae* and *Bacteriovoracaceae* at 200 m (*p*-value = 1). In Lake Annecy (Figure 2B), mean abundances at 3 and 45 m were not significantly different for *Bdellovibrionaceae* and *Bacteriovoracaceae* (*p*-value = 0.4357 and 0.3572) but they were for *Peredibacteraceae* (*p*-value = 0.03407). The mean abundance at 3 m was significantly different between the different BALOs (*p*-value = 0.00357), in particular between *Bacteriovoracaceae* and *Peredibacteraceae* (*p*-value = 0.00314) and between *Bacteriovoracaceae* and *Bdellovibrionaceae* (*p*-value = 0.04410). The mean abundances at 45 m were all significantly different between the three families (*p*-value = 0.000007), i.e., between *Bdellovibrionaceae* and *Bacteriovoracaceae* (*p*-value = 0.0150), *Bdellovibrionaceae* and *Peredibacteraceae* (*p*-value = 0.0334), and between *Bacteriovoracaceae* and *Peredibacteraceae* (*p*-value = 0.00002).

### 3.2. Relationships between BALOs Abundance and Environmental Variables

**Figure 3:**
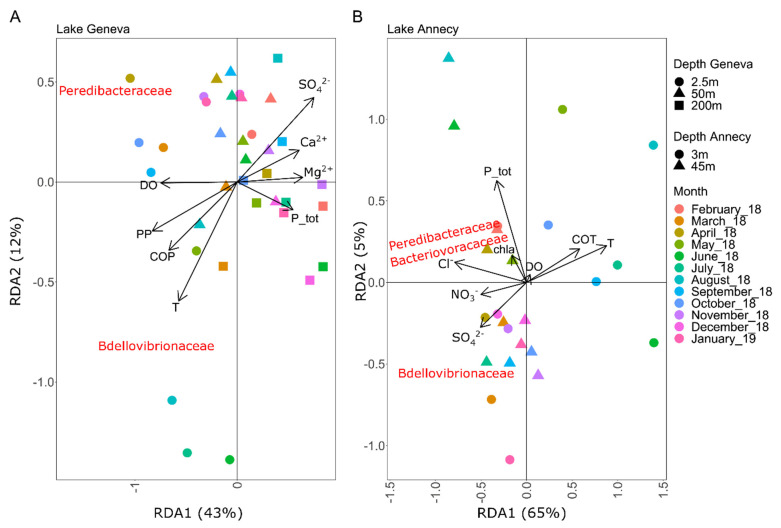
RDA triplots showing the relationship between BALOs abundance and significant environmental variables (VIF < 10) in Lakes Geneva and Annecy. In Lake Geneva (**A**), the first and second axis explained 43 and 12% of the variability, respectively (Anova *p*-value = 0.001). In Lake Annecy (**B**), the first and second axis explained 65 and 5% of the variability, respectively (Anova *p*-value = 0.001). Arrows indicate the direction and magnitude of variables. Ca^2+^: Calcium ion; chla: Chlorophyll a; Cl^−^: chloride; DO: Dissolved Oxygen; COP: Particulate Organic Carbon; COT: Total Organic Carbon; Mg^2+^: Magnesium ion; NO_3_^−^: Nitrate; PP: Particulate Phosphorus; P_tot: Total Phosphorus; SO_4_^2−^: Sulfate ion; TAC: Water Hardness; T: Temperature.

The first and second axis of the RDA explained for Lakes Geneva (Figure 3A) and Annecy (Figure 3B) 43% and 12%, and 65% and 5% of the total variability, respectively. In Lake Geneva, *Bdellovibrionaceae* were positively related to high values of temperature. *Peredibacteraceae* variability was associated with high dissolved oxygen and low total phosphorus concentrations. In Lake Annecy, *Bdellovibrionaceae* were related to high concentrations of sulfate while *Peredibacteraceae* and *Bacteriovoracaceae* were positively related to high concentrations of chlorine and chlorophyll a.

### 3.3. OTUs Diversity and Structure

We captured the diversity of *Bdellovibrionaceae* and *Peredibacteraceae* in Lake Geneva at 2.5 and 200 m, and in Lake Annecy at 3 and 45 m. Here, due to difficulty in amplifying *Bacteriovoracaceae* DNA we chose not to further investigate their diversity data. We obtained 110 OTUs for *Bdellovibrionaceae* and 109 OTUs for *Peredibacteraceae*. Rarefaction curves (Figure S3) revealed that the number of OTUs did not reach a plateau. The OTUs of *Bdellovibrionaceae* and *Peredibacteraceae* with higher number of reads (i.e., most abundant OTUs in number) were both found in the two lakes. Overall, Lakes Geneva and Annecy shared 58 OTUs of *Bdellovibrionaceae* and 32 OTUs of *Peredibacteraceae* (Figure S4). Lake Geneva had more unique OTUs than Lake Annecy. Figure S4C,D reveals that, in Lake Geneva, the majority of unique *Bdellovibrionaceae* OTUs (i.e., 20 OTUs) were located in the bottom layer, whereas unique *Peredibacteraceae* OTUs (i.e., 30 OTUs) were present in the upper layer. In Lake Annecy, the majority of the unique *Peredibacteraceae* OTUs (i.e., 23 OTUs) were found at 45 m (Figure S4D). Phylogenetic trees for *Bdellovibrionaceae* and *Peredibacteraceae* (using either Maximum Likelihood or Bayesian inference) were reconstructed as described in the supplementary section (Box S1) using the reference sequences of BALOs listed in Table S2 of the same supplementary. *Bdellovibrionaceae* OTUs (Figure S4) clustered closer to *Bdellovibrio* reference sequences, namely, *B. bacteriovorus* (e.g., OTUs 12, 21, 42, 47 and 194) and *B. exovorus* (e.g., 4, 8, 24, 46, and 83). The other *Bdellovibrionaceae* OTUs clustered far from the latter but not with other BALOs reference sequences. *Peredibacteraceae* OTUs (Figure S2) were closely related to *Peredibacter* namely *P. starrii*. OTUs composition for *Bdellovibrionaceae* and *Peredibacteraceae* were different from a lake to another as shown by the NMDS and Adonis test (NMDS: K = 2, stress = 0.13; Adonis: *p*-value = 0.003; Figure S7). However, OTUs composition was not significantly different when both depth and month were analyzed together, except for Lake Annecy for which the depth could be the reason behind a variation in OTUs composition (*p*-value = 0.30, 0.20, 0.04 and 0.78). *Bdellovibrionaceae* and *Peredibacteraceae* OTUs and environmental variables in Lake Geneva (Figure S8A) correlated positively (e.g., temperature (T) and ammonium (NH_4_^+^) and negatively (e.g., chloride (Cl^−^) and chlorophyll (chla)), but again, these relations were not significant. The same pattern was found for Lake Annecy OTUs (Figure S8B).

## 4. Discussion

BALOs are fascinating bacteria because they are the only known bacterial hunters with the obligation to find bacterial prey to grow and reproduce [5]. While their use in a variety of fields has been proposed, as potential or efficient biological-based therapeutic agents against bacterial pathogens [10], their ecology remains largely underexplored compared to other bacterial groups, especially in natural aquatic systems [38]. However, such microbial predation might be an essential biotic interaction in the maintenance of ecological balance [7,8]. Therefore, the aim of this study was to elucidate the abundance, distribution and diversity of some BALOs in the epilimnion and hypolimnion of two peri-alpine lakes. Note that we conducted this ecological study without taking into account biotic interactions with other microorganisms. In fine, this long-term study (over a year), which encompasses seasonality and different compartments of the water column, adds to our previous studies that investigated shorter term dynamics [18,24,25,39,40], and it allows for a better understanding of the ecology of *Bdellovibrionaceae*, *Peredibactearaceae* and *Bacteriovoracaceae* families in peri-alpine lakes.

The first important result is that we could find all targeted BALOs in the two lakes at different depths and months. Amongst them, some families reached relatively high concentrations, and various dynamics were recorded. This functional group of bacteria has been found in a wide variety of natural or man-made environments [41,42], and here, they probably take part in the ecological processes of the lakes. When comparing the two lakes, we observed that BALOs were rather concentrated in surface waters of Lake Geneva, whereas higher abundances were measured deeper in Lake Annecy. Overall, *Peredibacteraceae* were the most abundant BALO, with a preference for the upper layers in both lakes. Comparatively, *Bdellovibrionaceae* were globally more abundant at greater depth, especially in Lake Geneva. At last, whatever the system or depth examined, *Bacteriovoracaceae* were much less abundant than *Peredibacteraceae* and *Bdellovibrionaceae*. Such a distribution has already been observed [24] and suggests that each BALO type have a niche preference. Here, we only studied BALOs in the water column and it is possible that *Bacteriovoracaceae* prefer other habitats. Indeed, BALOs are generally more dominant in closed systems, sediments or biofilms compared to the open water [43,44]. For example, *Bacteriovoracaceae* are studied and isolated from sewage [38].

Three main peaks of BALOs’ abundance were observed, between March and April, between July and August, and between October and November. These observations are consistent with previous studies [24,25,43,44,45] and coincided with the peaks of the total bacteria measured by flow cytometry in this study from February to December (Figure S4). It is hypothesized here that this relationship could be attributed to favorable conditions, likely allowing efficient growth and development of the bacteria, both prey and predators, the latter depending on the former. It is noteworthy that these peaks of abundances were also observed at 45 m in Lake Annecy, but the dynamics were different than at the surface, perhaps because *Peredibacteraceae* were more abundant at depth in this lake. Moreover, the first peak observed at 3 m in Lake Annecy was observed in winter, highlighting again the capacity of these bacteria to develop and occupy very different niches and periods, as observed elsewhere, for instance, in arctic marine sediments [38]. It is also possible that BALOs, being active swimmers [46], can migrate from one layer to another where competition is lower and/or prey more available. Future studies are required to access the predation preference of BALOs towards heterotrophic and autotrophic bacteria that occupy distinct ecological niches. The correlation matrix from Figure S9A showed positive correlation between *Bdellovibrionaceae* and *Peredibacteraceae* abundance with bacterial abundance. No correlation was obtained for Lake Annecy (Figure S9B). It is noteworthy that when prey is absent or limited, BALOs may deplete rapidly and survival becomes compromised [47,48]. The low abundance of bacteria in Lake Annecy, which is an oligotrophic lake, may explain the low abundance and diversity of BALOs in it compared to Lake Geneva (mesotrophic state). As already suggested (e.g., [49,50]), there is possibly a minimal amount of prey needed to prevent predator decline. It is thus assumed that at periods where low abundances were recorded for the different BALOs, prey were likely insufficient to sustain the predators, whereas when peaks were recorded for one of the BALO family or another, this may be because cells rapidly responded to prey availability. Recently, Sathyamoorthy et al. [48] showed that *B. bacteriovorous* is indeed altered (with declining swimming capacities) when starved but also that this species can recover rapidly its capacity upon the introduction of prey. More generally, despite preying on a wide range of bacteria, it is likely that each BALO have different effects on various hosts in such mixed microbial assemblages, typical of natural ecosystems. Overall, biotic interaction data with prey and other microorganisms are crucial to better understand the determinants of BALOs abundance and distribution, since they are obligate predators [6]. Shotgun metagenomics sequencing, co-occurrence network, and microcosms set up with prey and others microorganism could shed more light on these mechanisms. We partially performed such experiments in other studies [25,40] and found that BALOs such as *Peredibacteraceae* are positively correlated to bacteria with high DNA content, in other word containing more nutrients. However, we did not find any relation with virus-like particles. In addition, we statistically linked *Bdellovibrionaceae* to other Gram-negative bacteria that could serve as prey. That being said, the information is still partial and we need, in a future study, to dedicate exclusively experiments for the biotic aspects, e.g., isolate prey, isolate BALOs, isolate BALOs predator and competitor, assess all environmental bacteria via metabarcoding, functional profile with shotgun sequencing and use qPCR to quantify BALOs prey, competitor and predator. Another piece of information in our study that directs towards biotic interaction is the difference in behavior from *Peredibacteraceae* in Lake Geneva and Annecy, suggesting a species-specific response that shapes the families’ responses to the environment, or that top-down regulation is much more important than bottom-up controls (and since BALOs are predators, it is highly probable that this is the case).

No strong relationships were found between environmental factors and the distribution of BALOs. We assume that since BALOs are predators, it is more likely that they are mainly associated with their prey, themselves more dependent on environmental descriptors. Nevertheless, we could report that temperature was likely important to explain *Bdellovibrionaceae* abundances in Lake Geneva. In fact, the abundance of studied BALOs increased in warmer months such as July and August. Other studies have shown the importance of temperature on the growth and abundance of BALOs [44,51]. Here, the temperature varied between 5 °C and 25 °C in Lakes Geneva and Annecy. The temperature could directly affect BALOs by acting on its growth or indirectly by acting on their prey. Low variations in temperature (4 ± 1 °C) such as in the study of Kandel et al. [43] in aquaculture ponds were suggested not to affect BALOs abundances. It takes large temperature fluctuations, as in natural systems such as here, to observe an effect on BALOs. Most surprisingly, the potential relationship of chloride (known to be a biocide at high levels) and chlorophyll a (autotrophic microorganisms) with *Peredibacteraceae* and *Bacteriovoracaceae* in Lake Annecy would deserve confirmation using laboratory experiments. A study reported that chloride ions are not necessary for growth in 44 different bacterial strains, except in very salty environments [52]. Naturally, this is not the case for freshwater lakes, and the chloride level only varied by 0.9. On the other hand, Huang and Starr [53] reported that some cations, magnesium and calcium, could play a role in predator–prey interaction. In the literature, other factors such as pH have been reported to affect BALOs predation. *Bdellovibrio* sp. motility was shown to be inhibited at pH < 5 and pH > 9 [54,55]. In this study, pH in both lakes only varied by 1, from 7.8 to 8.8. Globally, both the variability of BALOs’ abundance and distribution was poorly explained by environmental variables, as already mentioned before [18,43]. Again, it will be also informative to conduct some predator–prey experiments with autotrophic bacteria and microalgae.

Based on the OTUs identified using Swarm-v2 algorithm [30], *Peredibacteraceae* and *Bdellovibrionaceae* were found to be relatively diverse, as shown in a previous study [18,25]. When looking at the OTUs shared and unshared between the two lakes, the dominant OTUs, with a high number of reads for *Bdellovibrionaceae* and *Peredibacteraceae* were shared, while OTUs with a low number of reads were not and most likely affiliated to each environment. Most unique OTUs for *Bdellovibrionaceae* were found at 200 m in Lake Geneva, while *Peredibacteraceae* most unique OTUs were found at 2.5 m in Lake Geneva and 45 m in Lake Annecy. We therefore hypothesize that some BALOs can adapt to different environments or are tolerant to environmental variations (i.e., genetic adaptation), while others are only adapted to the environment in which they are located (i.e., environmental preference). Finally, the presence of *Peredibacteraceae* and *Bdellovibrionaceae* in the lacustrine environments (and other environments, e.g., sea water) aroused our interest in studying their genetic structure in order to uncover the mechanisms that allow them to exist in different environments. New mechanisms have been revealed for heterotrophic bacteria such as *Escherichia coli*, via a remodeling of their gene expression through, e.g., transcriptional and post-transcriptional regulation [56]. However, can it apply in an obligate predatory bacteria? It is also well known that some species of microalgae exhibit different biogeography granted by genomic adaptation, i.e., in the psychrotolerant *Stichococcus* spp. [57]. The phylogenetic trees suggested that *Bdellovibrionaceae* OTUs diversity is much higher than *Peredibacteraceae* OTUs that cluster close to *P. starrii*. *Bdellovibrionaceae* OTUs clustered with *B. bacteriovorus* and *B. exovorus* strains, but many others are distant suggesting that there is a rich diversity of *Bdellovibrionaceae* yet to be uncovered. Despite being less diverse, *Peredibacteraceae* are more abundant, suggesting that they have adapted efficiently to peri-alpine lakes. References [58,59,60,61,62,63,64] are cited in the supplementary materials.

The most important questions are still pending: what is the role of these bacteria, and to what extent do they influence the composition and dynamics of the bacterial community? The next step will be to perform dedicated experiments to quantify BALOs’ predation, to assess how it is comparable to other biotic pressure (e.g., viral lysis, flagellate or ciliate grazing) and how it can influence the carbon cycle in aquatic ecosystems. 

## 5. Conclusions

The present study supported that BALOs are present and sometimes relatively abundant and diverse in lakes, where they occupy diverse ecological niches. Among them, *Peredibacteraceae* was the dominant group, likely driven by biotic variables and interactions, which was not investigated here. The high abundance of *Peredibacteraceae* and the high diversity of *Bdellovibrionaceae* suggest different ecological impacts on bacterial population that need to be investigated.

## Data Availability

The raw files (R1 and R2), tags list, unfiltered and filtered OTUs table of the run datasets obtained in this study have been deposited at Zenodo’s depository under https://doi.org/10.5281/zenodo.4293824, accessed on 23 march 2020. In addition, most of the OTU sequences used to construct the phylogenetic tree can be found at NCBI GenBank under accession numbers: MW299511:MW299708 and MW302902:MW302988.

## Supplementary Materials

The following supporting information can be downloaded at: https://www.mdpi.com/article/10.3390/microorganisms10101960/s1, Figure S1: Sum of BALOs abundances in copy per mL in Lakes Geneva and Annecy for all sampled depths and months. Table S1: BALOs abundances in copy per mL in the two studied ecosystems for all the sampled depths throughout the year. Figure S2: One-year distribution and dynamics of Bdellovibrionaceae, Peredibacteraceae, and Bacteriovoracaceae in the water column at three different depths sampled in Lakes Geneva and Annecy. Figure S3: Rarefaction curve computed from BALOs OTUs obtained after sequencing. Figure S4: Venn diagram showing shared and unshared OTUs in Lake Geneva and Annecy. Box S1: Phylogenetic trees were constructed using BALOs OTUs obtained by specific set of primers and BALOs references sequences. Table S2: Accession numbers of BALOs and other bacteria sequences downloaded from NCBI used to build the phylogenetic trees. Figure S5: Phylogenetic tree of *Bdellovibrionaceae* OTUs amplified with Bd F186-R481 in Lakes Geneva and Annecy. Figure S6: Phylogenetic tree of Peredibacteraceae OTUs amplified with Per F1024-R1349 in Lakes Geneva and Annecy. Figure S7: Nonmetric Multidimensional Scaling (NMDS) showing the placement of the samples from the Miseq run (*Bdellovibrionaceae* and *Peredibacteraceae*) in the ordination space (stress = 0.13) with 95% ellipses. Figure S8: Canonical Correspondence Analysis (CCA). Ordination diagram of the BALOs community data with environmental variables. The CCA model of Lakes Geneva and Annecy are not significant according to ANOVA. Figure S9: Evolution of the prokaryote-like particle abundances in Lakes Geneva and Annecy in the water column from January to December 2018 as measured by flow cytometry. Gaps represent missing measurements. Figure F2: Spearman’s rank correlation test was conducted to examine the correlation between BALOs and prokaryotes abundances. Matrixes of correlation were drawn following Antoine Soetewey’s code at “https://statsandr.com/blog/correlation-coefficient-and-correlation-test-in-r/ (accessed on 23 March 2020)”. The correlograms showed the correlation coefficient for all pairs of variables (with more intense colors for more correlations), and correlations not statistically significant are represented by a white-box.
