# Supplementary material for: A Comparative Study of the Dynamics and Diversity of Bdellovibrio and Like Organisms in Lakes Annecy and Geneva
Source: Microorganisms. 2022 Sep 30;10(10):1960. doi: 10.3390/microorganisms10101960 (PMC9610775; doi:10.3390/microorganisms10101960)
Supplement: Supplementary file 1 [file microorganisms-10-01960-s001.zip › microorganisms-1904465-supplementary.pdf]

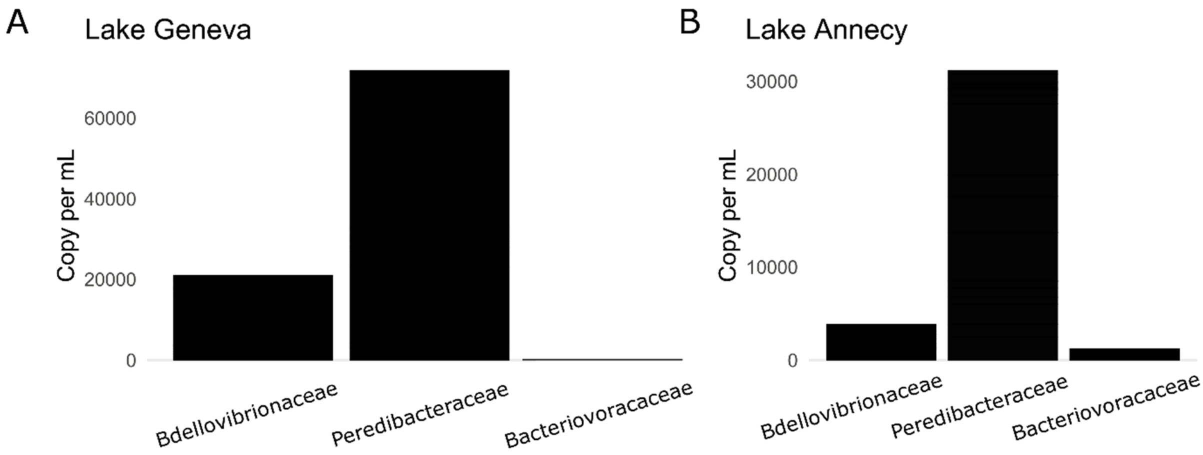

Figure S1: Sum of BALOs abundances in copy per mL in Lakes Geneva and Annecy for all sampled depths and months. The barplots show that *Peredibacteraceae* dominated in number of 16S rDNA copies in the two studied ecosystems.

Table S1: BALOs abundances in copy per mL in the two studied ecosystems for all the sampled depths throughout the year. Minimal, maximal and mean values that are highlighted in gray were calculated with missing values.

	**Lake Geneva ($Copy\ mL^{-1}$)**			**Lake Annecy ($Copy\ mL^{-1}$)**		
	Min	Max	Mean	Min	Max	Mean
Bdellovibrionaceae	30	11,858	582	3	598	161
Peredibacteraceae	5	37,270	1,992	6	7,604	1,297
Bacteriovoracaceae	3	310	45	1	655	52

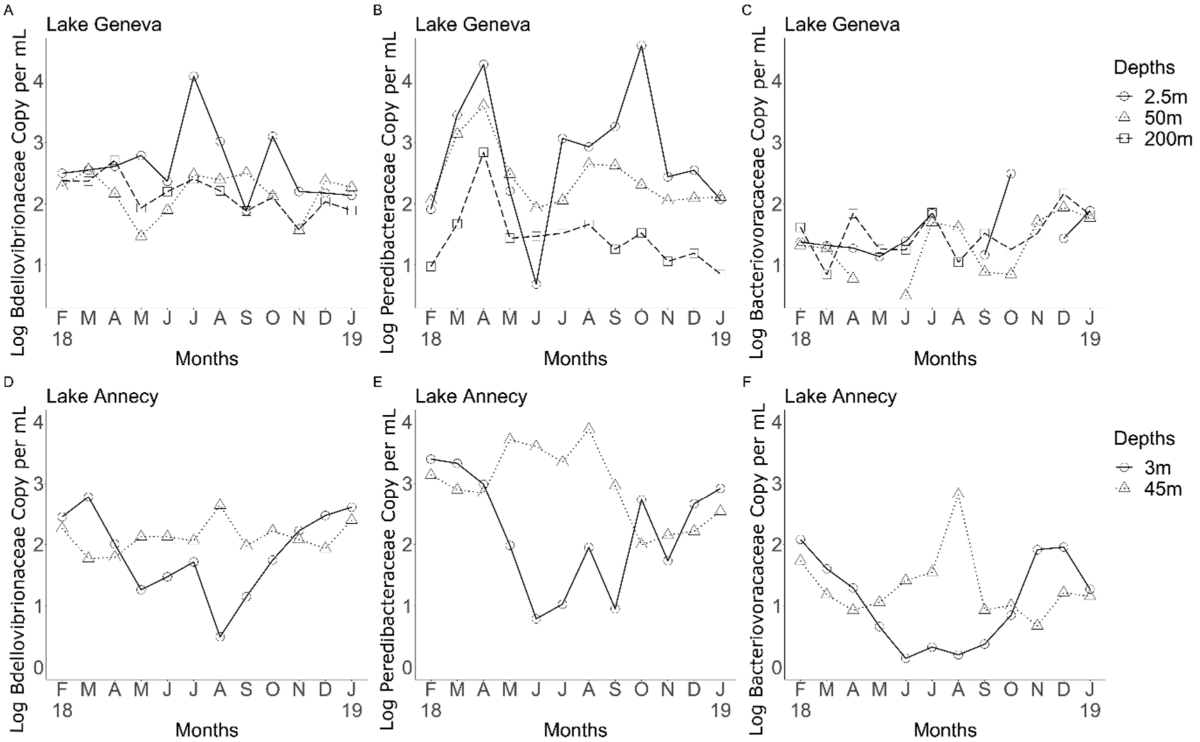

Figure S2 One-year distribution and dynamics of *Bdellovibrionaceae*, *Peredibacteraceae*, and *Bacteriovoracaceae* in the water column at three different depths sampled in Lakes Geneva and Annecy.

F: February; M: March; A: April; M: May; J: June; J: July; A: August; S: September; O: October; N: November; D: December; J: January.

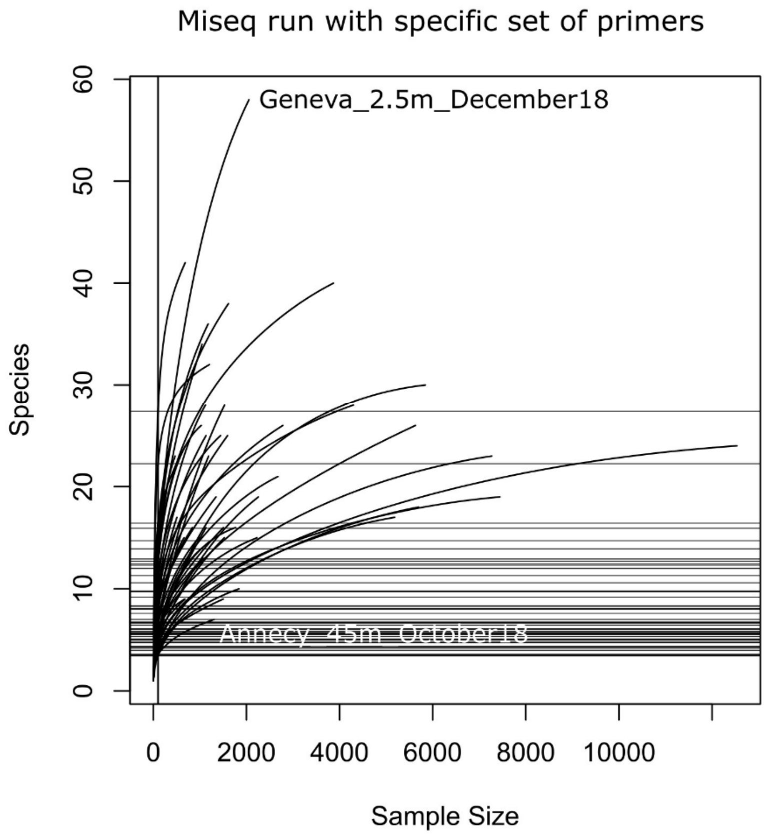

Figure S3: Rarefaction curve computed from BALOs OTUs obtained after sequencing.

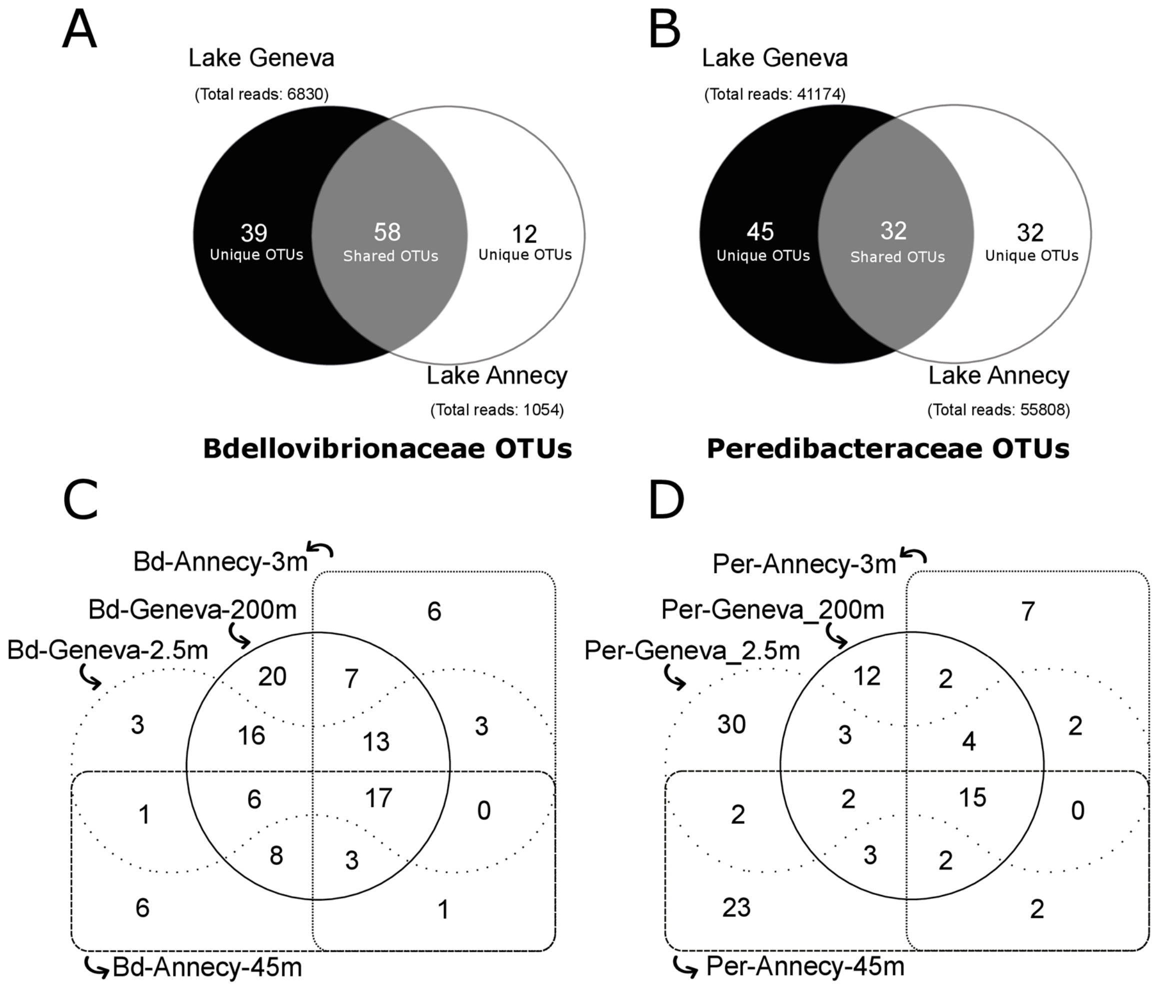

Figure S4: Venn diagram showing shared and unshared OTUs in Lake Geneva and Annecy. (A) symmetric Venn diagrams of *Bdellovibrionaceae* OTUs in the two lakes for all sampled depths. (B) symmetric Venn diagrams of *Peredibacteraceae* OTUs in the two lakes for all sampled depths (Filled black circle represent Lake Geneva unique OTUs; Filled grey circle: Intersection of shared OTUs between the two lakes; Filled white circle represent Lake Annecy unique OTUs). (C) asymmetric Venn diagrams showing shared *Bdellovibrionaceae* OTUs between Lake Geneva and Annecy in the surface and the bottom. (D) asymmetric Venn diagrams showing shared *Peredibacteraceae* OTUs between Lake Geneva and Annecy in the surface and at the bottom.

Box S1:

Phylogenetic trees were constructed using BALOs OTUs obtained by specific set of primers and BALOs references sequences (Table S1). Briefly, OTUs and reference sequences were first aligned and visualized using MUSCLE [59] and MEGA X [60]. Then, sequences were trimmed to obtain equal length. The alignment generated respectively 229 and 235 positions for *Bdellovibrionaceae* and *Peredibacteraceae.* The alignments were curated with Gblocks 0.91b [61], yielding respectively 221 and 268 positions. ModelGenerator v.85 [62] was used to select the best nucleotide substitution model with discrete gamma categories set to 4. Akaike information criterion (AIC) [63] was used for model selection. The models used to compute *Bdellovibrionaceae* and *Peredibacteraceae* trees were respectively GTR + G (0.29) and K80 + G (1.11). The trees were then built using Maximum likelihood (100 bootstrap replicates) with PhyML 3.1 [64] and Bayesian method (1 to 1.5 million generations and 25 % burn-in value) using MrBayes 3.2.7a [65].

Table S2: Accession numbers of BALOs and other bacteria sequences downloaded from NCBI used to build the phylogenetic trees.

Sequence name	Accession number
Bdellovibrio bacteriovorus strain HD127	AJ29276.1
Bdellovibrio sp. W strain ATCC 27047	AJ292518.1
Bdellovibrio bacteriovorus strain 109J	M61234.1
Bdellovibrio bacteriovorus strain HD100	NR027553.1
Bdellovibrio exovorus strain JSS	EF687743.1
Peredibacter sp. Uncultured clone 1	KT308262.1
Peredibacter sp. Uncultured clone 2	JX525305.1
Peredibacter sp. Uncultured clone 3	KC545751.1
Peredibacter sp. Uncultured clone 4	JX525192.1
Peredibacter starrii strain A3.12	NR024943.1
Bacteriovorax stolpii strain Uki2	NR115142.1
Bacteriovorax sp. strain F2	AY294218.1
Micavibrio aeruginosavorus strain ARL-13	DQ186612.1
Micavibrio sp. strain EPB	DQ186613.1
Pseudobacteriovorax antillogorgiicola strain RKEM611	KJ685394.1
Halobacteriovorax marinus strain SJ	NR102485.1
Halobacteriovorax litoralis strain JS5	NR028724.1
Silvanigrella aquatica strain MWH-Nonnen-W8red	NR157754.1
Vampirovibrio chlorellavorus strain ICPB 3707	NR104911.1

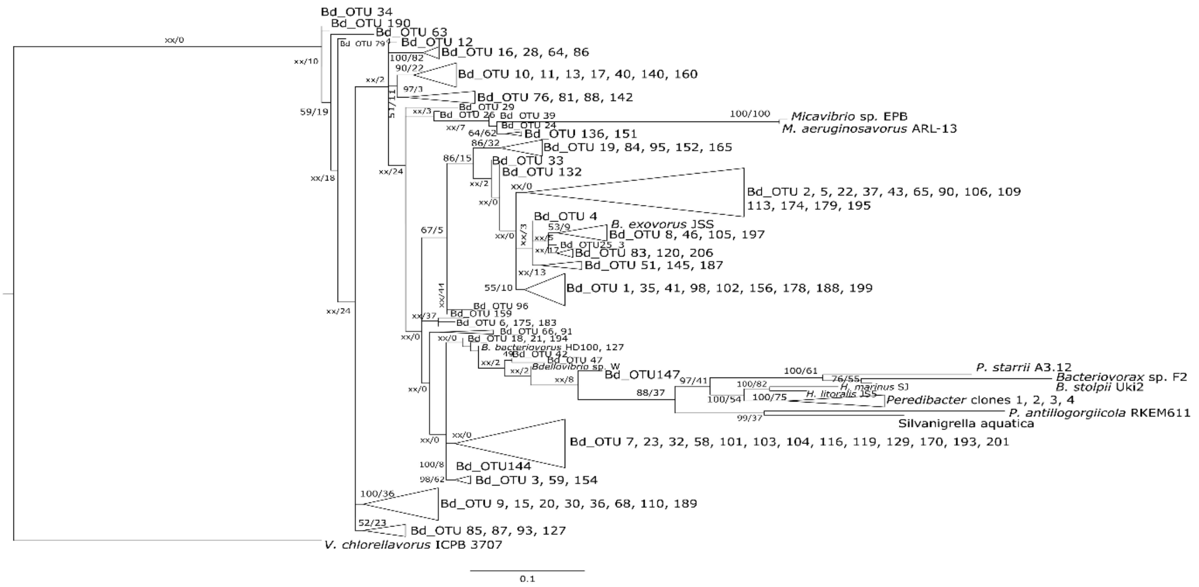

Figure S5: Phylogenetic tree of *Bdellovibrionaceae* OTUs amplified with Bd F186 - R481 in Lakes Geneva and Annecy. The tree was built using Maximum Likelihood and Bayesian methods. The alignment curated with Gblocks yielded 221 positions. The substitution model used is GTR + G (0.29). Posterior probabilities / bootstrap values are shown at each node when possible. The tree is rooted using *Vampirovibrio chlorellavorus*.

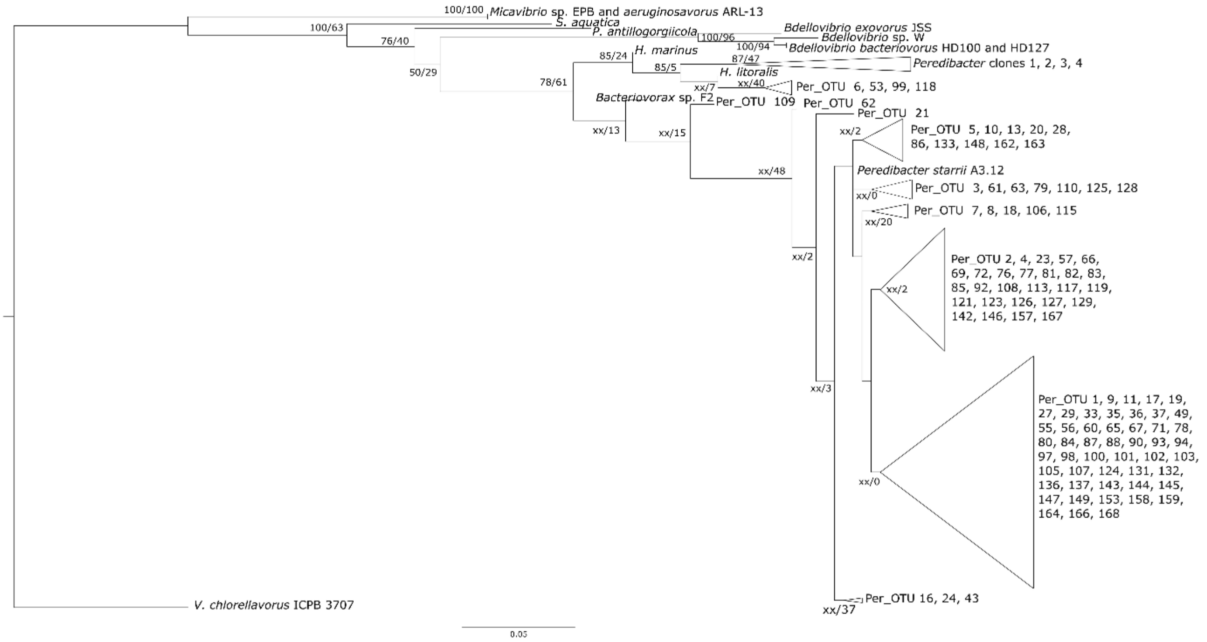

Figure S6: Phylogenetic tree of *Peredibacteraceae* OTUs amplified with Per F1024 – R1349 in Lakes Geneva and Annecy. The tree was built using Maximum Likelihood and Bayesian methods with the

evolutionary model K80 + G (1.11). The alignment curated with Gblocks yielded 268 positions. Posterior probabilities / bootstrap values are shown at each node when possible. The tree is rooted using the outgroup *Vampirovibrio chlorellavorus*. Most *Peredibacteraceae* OTUs are phylogenetically related to the type species *Peredibacter starrii* A3.12.

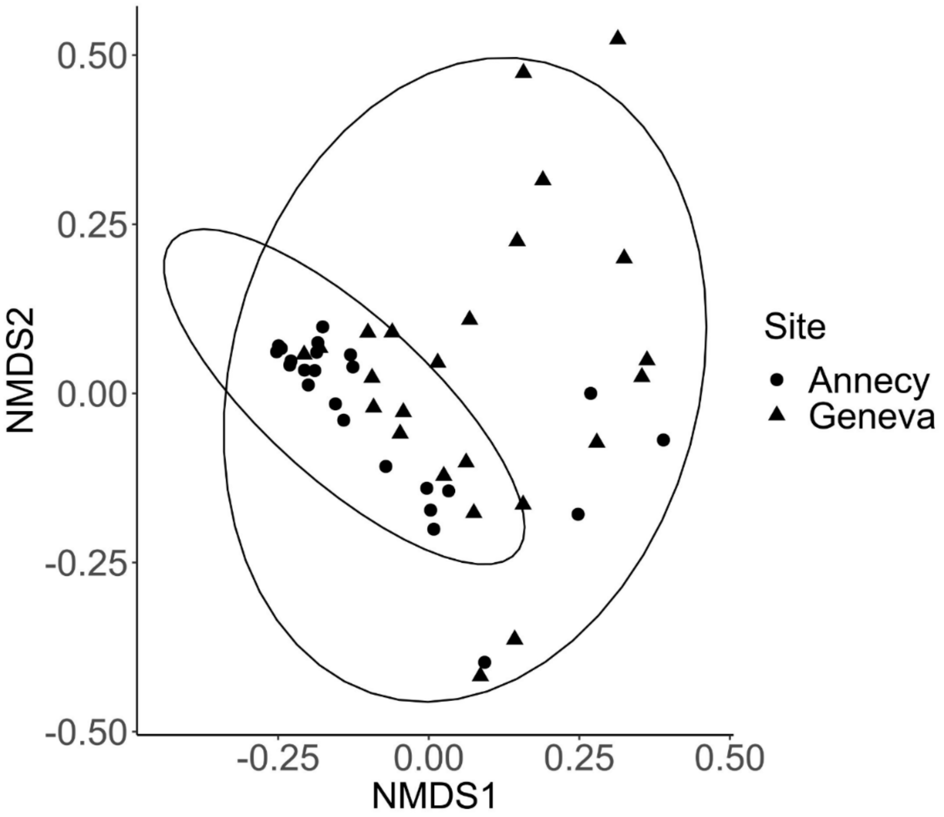

Figure S7: Nonmetric Multidimensional Scaling (NMDS) showing the placement of the samples from the Miseq run (*Bdellovibrionaceae* and *Peredibacteraceae*) in the ordination space (stress = 0.13) with 95% ellipses. Permutational Multivariate Analysis of Variance (Adonis) showed that OTUs are statistically different from each other between the two lakes (p-value = 0.003) and between the two sampled depths of Lake Annecy (p-value = 0.04).

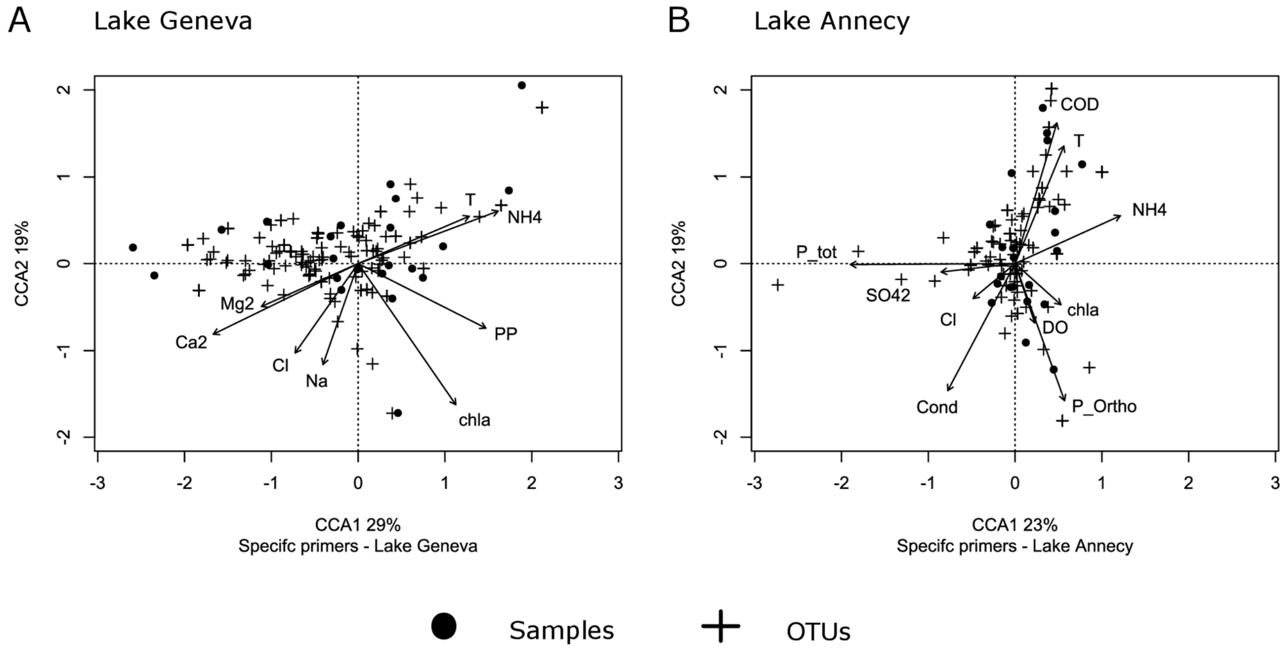

Figure S8: Canonical Correspondence Analysis (CCA). Ordination diagram of the BALOs community data with environmental variables. The CCA model of Lakes Geneva and Annecy are not significant according to ANOVA.

Ca2: Calcium ion; Mg2: Magnesium ion; Cl: chloride; Na: Sodium; chla: Chlorophyll a; PP: Particulate Phosphorus; NH4: Ammonium; P_tot: Total Phosphorus; SO42: Sulfate ion; Cond: Conductivity; P_Ortho: Orthophosphate ion; DO: Dissolved Oxygen; COD: Dissolved Organic Carbon; T: Temperature; NO2: Nitrite; SIOH4: Silicate; S: Salinity; PHEO: Phaeopigments; MES: Suspended particles.

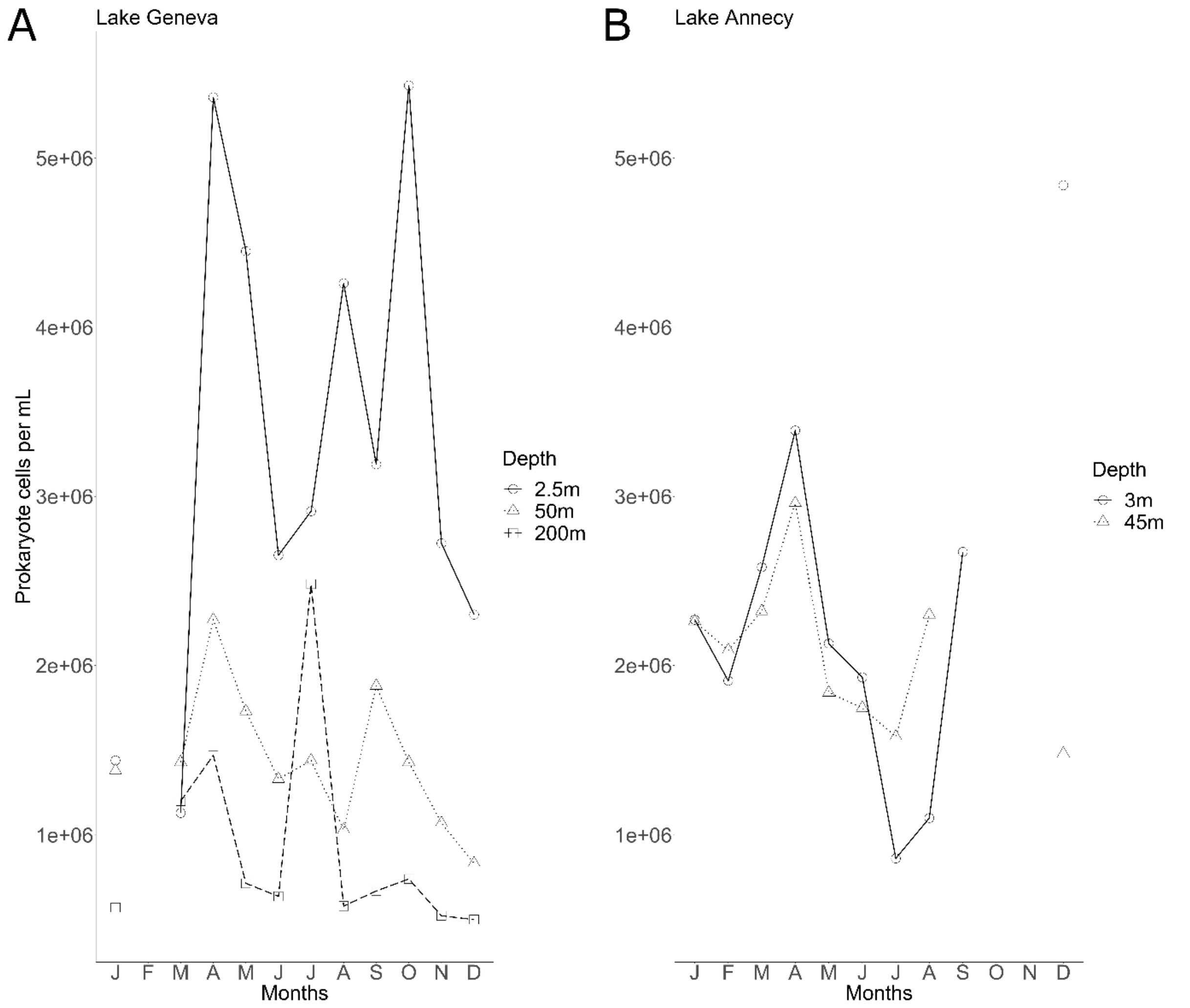

Figure S9: Evolution of the prokaryote-like particle abundances in Lakes Geneva and Annecy in the water column from January to December 2018 as measured by flow cytometry. Gaps represent missing measurements.

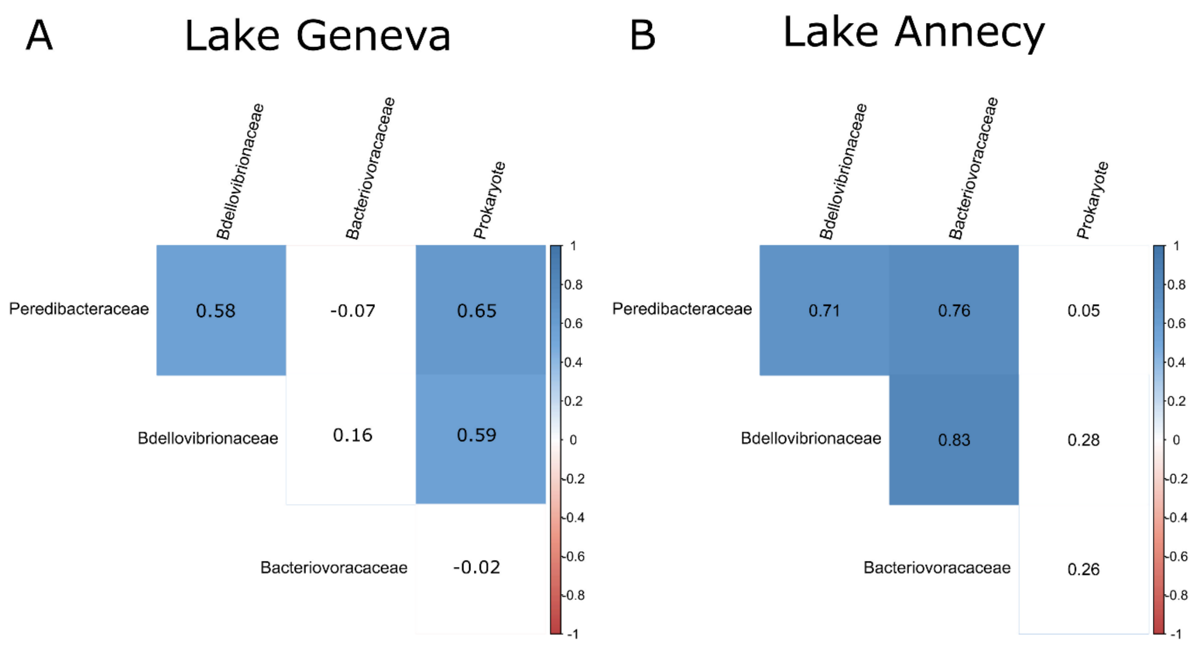

Figure F2: Spearman's rank correlation test was conducted to examine the correlation between BALOs and prokaryotes abundances. Matrixes of correlation were drawn following Antoine Soetewey's code at "https://statsandr.com/blog/correlation-coefficient-and-correlation-test-in-r/". The correlograms showed the correlation coefficient for all pairs of variables (with more intense colors for more correlations), and correlations not statistically significant are represented by a white-box.
